# DOCUMENTING THE VULNERABILITIES OF SELF-EMPLOYED OLDER ADULTS: AN EXAMPLE FROM THE PANDEMIC

**DOI:** 10.1093/geroni/igae098.1852

**Published:** 2024-12-31

**Authors:** Cal Halvorsen, Christina Matz, Bruna Lopez

**Affiliations:** Washington University in St. Louis, St. Louis, Missouri, United States; Boston College, Chestnut Hill, Massachusetts, United States; Boston College, Chestnut Hill, Massachusetts, United States

## Abstract

A defining factor of self-employment in later life is its autonomy and flexibility, enabling older workers to be their own bosses, set their own schedules, and determine where and how they work. Although self-employment has been linked with both positive (higher quality of life and health) and negative (lower take-home pay and workplace-provided benefits) outcomes, little research has considered how self-employed older workers fared during the COVID-19 pandemic. In response, we analyzed data from more than 9,000 Americans ages 50 and older from the 2020 Health and Retirement Study (HRS) and the 2021 HRS Perspectives on the Pandemic mail-in survey. We assessed how work status in 2020 (working for someone else, self-employed, both working for someone else and self-employed, or not working) related to financial difficulties and experiencing health problems due to missed or delayed healthcare outcomes in 2021. While controlling for sociodemographic and health variables, our two multivariable logistic regressions found that self-employment was significantly associated with worse outcomes. Specifically, those who were self-employed or self-employed and working for someone else had significantly higher odds of facing financial difficulties and health problems due to delayed or missed care during the pandemic than those working for someone else only. (Those not working had similar odds of experiencing these issues as those in wage-and-salary work.) This study highlights the vulnerabilities faced by many self-employed older adults while encouraging a discussion on how to better support their health and well-being.

